# Blood nesfatin-1 levels in patients with polycystic ovary syndrome: a systematic review and meta-analysis

**DOI:** 10.3389/fendo.2023.1275753

**Published:** 2024-01-24

**Authors:** Mei Wang, Jiao Tong, Qing Zhu, Huaiyun Tang, Lisha Tang

**Affiliations:** Clinical Centre of Reproductive Medicine, Lianyungang Maternal and Child Health Hospital, Lianyungang, Jiangsu, China

**Keywords:** meta-analysis, nesfatin-1, polycystic ovary syndrome, serum, systematic review

## Abstract

**Background:**

Previous studies have investigated the relationship between nesfatin-1 level and polycystic ovary syndrome (PCOS). However, these studies have produced conflicting results. Thus, in this meta-analysis, we aimed to clarify the association between blood nesfatin-1 levels and PCOS, and the ability of nesfatin-1 as a biomarker in PCOS.

**Methods:**

Meta-analysis was performed using STATA 12.0 software. We computed standard mean difference (SMD) and 95% confidence interval (CI) regarding the comparison of blood nesfatin-1 in patients with PCOS and controls.

**Results:**

The present meta-analysis showed no significant difference in blood nesfatin-1 level between patients with PCOS and controls with a random effects model (SMD = 0.03; 95%CI: -0.71, 0.77; I^2 =^ 97.1%, *p* value for Q test < 0.001). Subgroup analysis for different ethnicities reported no significant difference in blood nesfatin-1 level between patients with PCOS and controls in both Caucasian and Asian populations. Subgroup analysis for different sample types reported no significant difference in serum nesfatin-1 level between patients with PCOS and controls. Subgroup studies reported no significant difference in blood nesfatin-1 level between PCOS and controls in both obese and non-obese populations.

**Conclusion:**

In conclusion, there is no significant relationship between blood nesfatin-1 levels and PCOS.

## 1 Introduction

Polycystic ovary syndrome (PCOS) is one of the most common female endocrine disorders without exact etiology currently, affecting approximately 6%-10% of women worldwide (1). PCOS patients are most characterized by sex hormone imbalance, with hallmark features of acne, hirsutism, infertility, irregular menstrual cycle, and polycystic appearing ovaries on ultrasound (2). The Rotterdam diagnostic criteria for PCOS are now internationally endorsed and are based on two of three features: oligo- or anovulation, hyperandrogenism (clinical or biochemical), and polycystic ovaries (3). Additionally, the evidence indicates that PCOS is associated with several endocrine and metabolic disorders, including insulin resistance, and dyslipidemia (4, 5). A recent narrative review proposed that the levels of nesfatin-1, myonectin, omentin, and neudesin were decreased in PCOS patients, while the levels of the other considered agents (e.g., preptin, gremlin-1, neuregulin-4, xenopsin-related peptide, xenin-25, and galectin-3) were increased (6).

Nesfatin-1 is widely expressed in both the central nervous system and peripheral tissue with the role of regulating metabolism, appetite, gut motility, and feeding behavior (7, 8). As a multifunctional biomolecule, nesfatin-1 plays an important role in the diagnosis and treatment of many diseases, including coronary artery disease (9), multiple sclerosis (10), type 2 diabetes mellitus (11). Studies have shown that nesfatin-1 is related to the inhibition of lipid-related diseases, because it can reduce fat accumulation and increase lipid decomposition in the lipid metabolism (12).

As a newly discovered cytokine in 2006, previous studies have investigated the relationship between nesfatin-1 level and PCOS. However, these studies have produced conflicting results. Some studies revealed higher levels of nesfatin-1 in patients with PCOS relative to healthy controls, while others reported opposite findings. Thus, in this meta-analysis, we aimed to clarify the association between blood nesfatin-1 levels and PCOS, and the ability of nesfatin-1 as biomarker in PCOS.

## 2 Methods

This meta-analysis was performed according to the Preferred Reporting Items for Systematic Reviews and Meta-Analyses (PRISMA 2020) guidelines (13) and Meta-analyses of Observational Studies in Epidemiology (MOOSE) guidelines (14).

### 2.1 Literature search

Two reviewers (MW and JT) independently searched these databases (PubMed, Web of Science, EMBASE, Medline and Google Scholar) from the inception of the databases to June 30, 2023. We only included studies written in English. The search terms were (“nesfatin-1” OR “nesfatin” OR “markers” OR “biomarkers”) AND (“polycystic ovary syndrome” OR “PCOS”). Articles were discussed and decided by the three authors (MW, JT and QZ) after the appearance of inconsistent selections.

### 2.2 Study selection

Inclusion criteria: 1) study investigated blood nesfatin-1; 2) study investigated PCOS; 3) study written in English; 4) studies used control group. The control group had no clinical or biochemical evidence of PCOS.

Exclusion criteria: 1) reviews, meta-analysis and case reports; 2) letters book chapters, animal studies and published abstracts; 3) study which did not provide sufficient information about blood nesfatin-1 level in PCOS.

### 2.3 Data extraction

Two reviewers screened titles and abstracts of all articles. We extracted these data from included articles: first author, publication year, country, sample size, mean age, body mass index (BMI), blood nesfatin-1 concentrations, sample type and detection method.

### 2.4 Statistical analysis

Meta-analysis was performed using STATA 12.0 software. We computed standard mean difference (SMD) and 95% confidence interval (CI) regarding the comparison of blood nesfatin-1 in patients with PCOS and controls. Heterogeneity across studies was explored with I^2^ and Q test. A random effects model was used for I^2^ ≥ 50% and *p* value for Q test ≤ 0.05. A fixed-effects model was used for I^2^ < 50% and *p* value for Q test > 0.05. Meta-regression analysis was adopted to investigate the source of heterogeneity. Subgroup studies for different ethnicities and different sample types were conducted to investigate the source of the heterogeneity. Obesity in adults was defined by the World Health Organization (WHO) (15) as BMI > 30kg/m² for obese. Subgroup studies depending on the presence and absence of obesity was conducted to investigate the source of the heterogeneity. Sensitivity analysis was adopted to evaluate the stabilization of meta-analysis. Begg’s test was adopted to evaluate publication bias.

## 3 Results

### 3.1 Characteristics of included studies

**Figure 1:**
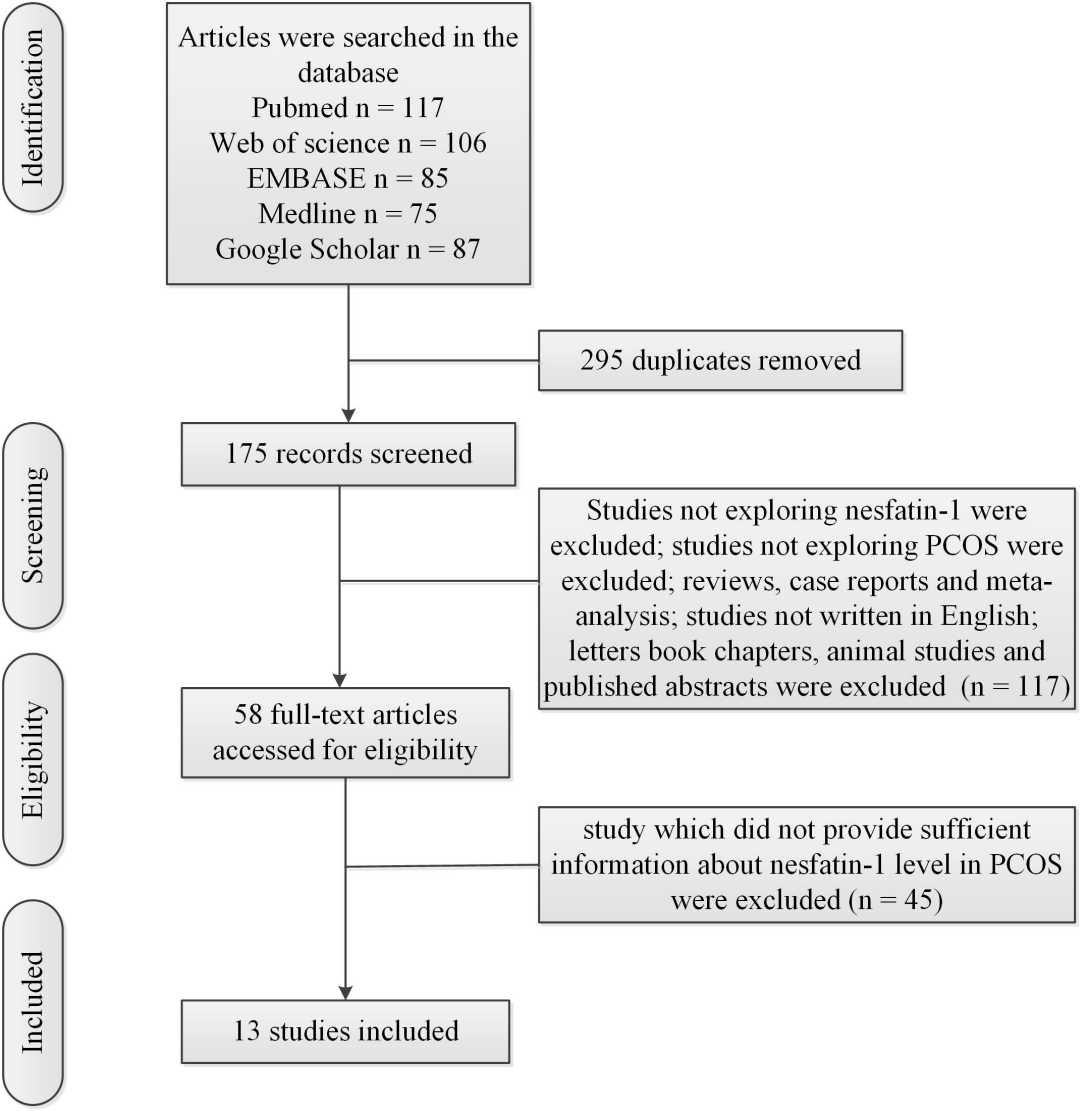
Flow chart of the literature search and selection process.

**Figure 1** showed the flow chart of the literature search and selection process. **Table 1** showed characteristics of included studies. Mean value and standard deviation (SD) of blood nesfatin-1 in patients with PCOS and controls were collected from 13 studies (16–28) (patients with PCOS: n = 757, controls: n = 569).

**Table 1:** Study characteristics of included studies.

Reference	Country	Subjects	Age (years)	BMI (kg/m^2^)	Results (mean ± SD)	Samples	Methods
Deniz et al. 2012 (16)	Turkey	Con: 30	23.16 ± 3.66	24.43 ± 0.50	2.22 ± 1.14 ng/mL	plasma	ELISA (Phoenix Pharmaceuticals)
		PCOS:30	23.56 ± 4.80	25.03 ± 0.86	0.88 ± 0.36 ng/mL		
Ademoglu et al. 2014 (17)	Turkey	Con: 28	26.2 ± 4.9	21.0 ± 2.8	275.55 ± 1.74 pg/mL	serum	ELISA (USCN Life Science)
		PCOS: 55	25.1 ± 5.6	27.4 ± 6.8	371.43 ± 2.50 pg/mL		
Binnetoğlu et al. 2014 (18)	Turkey	Con: 28	28 ± 6.17	22.81 ± 3.6	6.24 ± 3.69	plasma	ELISA (EIAab Science)
		PCOS: 37	25 ± 78	25.17 ± 4.9	6.56 ± 3.78		
Alp et al. 2015 (19)	Turkey	Con: 35	28.14 ± 6.766	22.34 ± 3.222	2.43 ± 0.846 ng/mL	serum	ELISA (Cloud-Clone)
		PCOS: 55	25.95 ± 5.612	24.03 ± 5.067	2.29 ± 0.558 ng/mL		
Sahin et al. 2015 (20)	Turkey	Con: 48	21.5 ± 4.5	29.7 ± 5.6	6.5 ± 2.9 ng/mL	serum	ELISA (USCN Life Science)
		PCOS: 54	22.2 ± 4.2	30.0 ± 7.5	10.2 ± 5.0 ng/mL		
Taskin et al. 2015 (21)	Turkey	Con: 26	26.85 ± 5.06	22.16 ± 2.47	154262700.5 ± 100199116.3 ng/mL	serum	ELISA (SunRed Biotechnology)
		Obese PCOS: 28	25.61 ± 4.58	35.81 ± 4.60	70015207.1 ± 46135532.1 ng/mL		
		Non-obese PCOS: 32	24.72 ± 4.30	23.83 ± 3.55	89883096.7 ± 49192130.5 ng/mL		
Ali et al. 2021 (22)	Iraq	Con: 40	29.5 ± 5.2	29.3 ± 5.1	6.3 ± 3.0 ng/mL	serum	ELISA
		PCOS: 45	29.3 ± 5.7	30.1± 4.2	11.1± 3.5 ng/mL		
Demir Caltekin et al. 2021 (23)	Turkey	Con: 40	28.23 ± 5.09	24.7 ± 3.7	36.8 ± 20.7 ng/mL	serum	ELISA ((Bioassay Technology Laboratory)
		PCOS: 44	26.41 ± 5.036	24.07 ± 2.97	17.08 ± 13.8 ng/mL		
Varli et al. 2021 (24)	Turkey	Con: 42	29.0 ± 3.7	23.7 ± 5.0	1830424848 ± 930447656.7 ng/mL	serum	ELISA (SunRed Biotechnology)
		PCOS: 41	27.7 ± 3.6	24.8 ± 4.2	1495249730 ± 877148222.1 ng/mL		
Wang et al. 2022 (25)	China	Con: 150	29 ± 12.1	NA	1.10 ± 0.97 mg/mL	serum	ELISA (NA)
		PCOS: 200	28.5 ± 10.1	NA	1.89 ± 0.99 mg/mL		
Hamed et al. 2022 (26)	Egypt	Con: 24	30.13 ± 3.26	25.43 ± 1.44	316.10 ± 59.87 pg/mL	serum	ELISA (Sinogeneclon Biotech)
		PCOS: 60	28.42 ± 4.34	31.32 ± 4.80	362.37 ± 85.06 pg/mL		
Salman et al. 2022 (27)	Iraq	Con: 48	28.12 ± 6.0	29.68 ± 4.7	0.858 ± 0.271 ng/ml	serum	NR
		PCOS: 46	27.23 ± 5.4	30.69 ± 3.1	0.439 ± 0.127 ng/ml		
Mahmood et al. 2023 (28)	Iraq	Con: 30	NR	NR	736.405 ± 259.222 pg/mL	serum	ELISA

BMI, body mass index; Con, control; ELISA, enzyme-linked immunosorbent assay; NA, not available; PCOS, polycystic ovary syndrome.

### 3.2 Meta-analysis results

**Figure 2:**
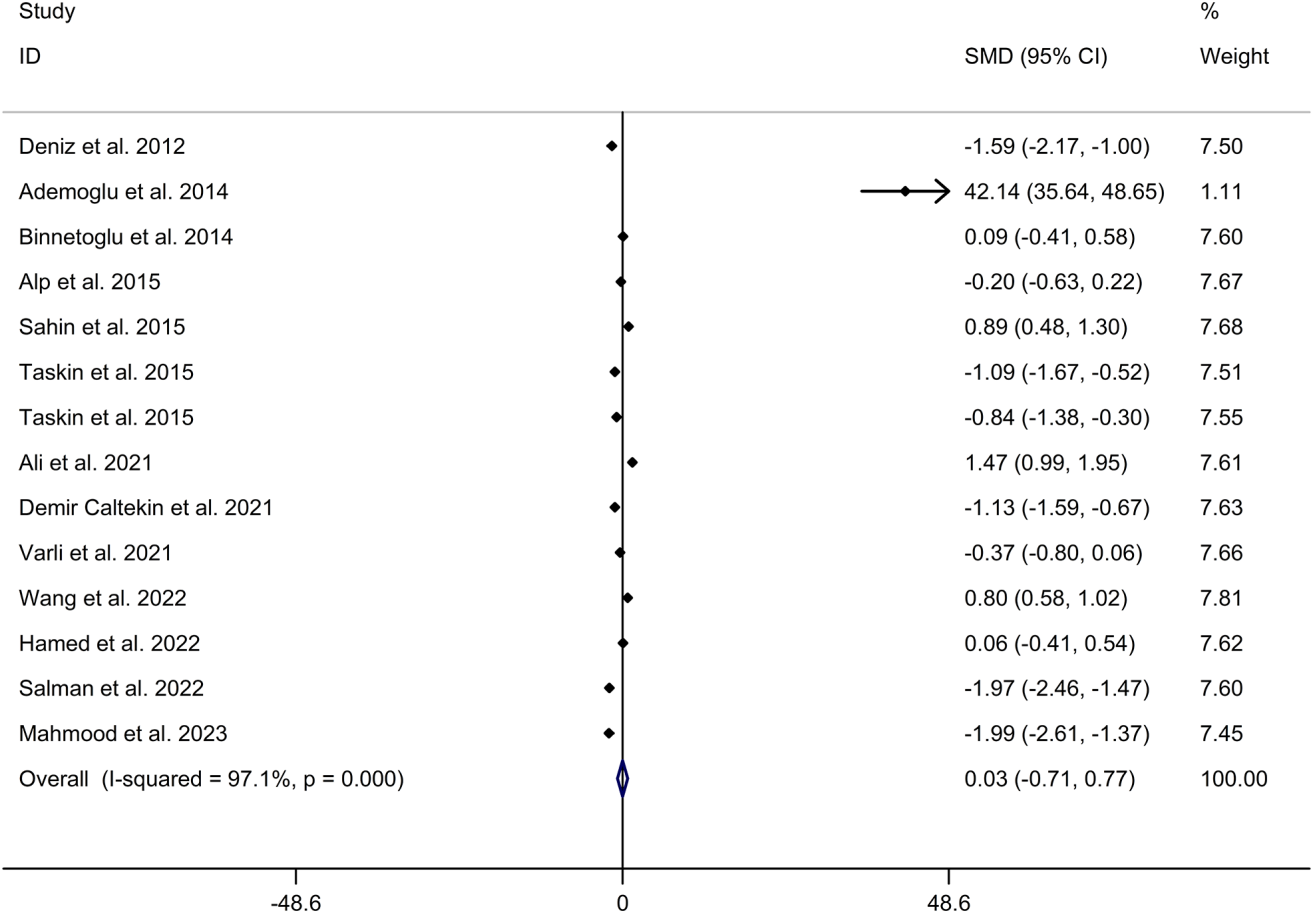
Forest plot for comparison in blood nesfatin-1 level between patients with PCOS and controls. CI, confidence interval; PCOS, polycystic ovary syndrome; SMD, standard mean difference.

**Figure 3:**
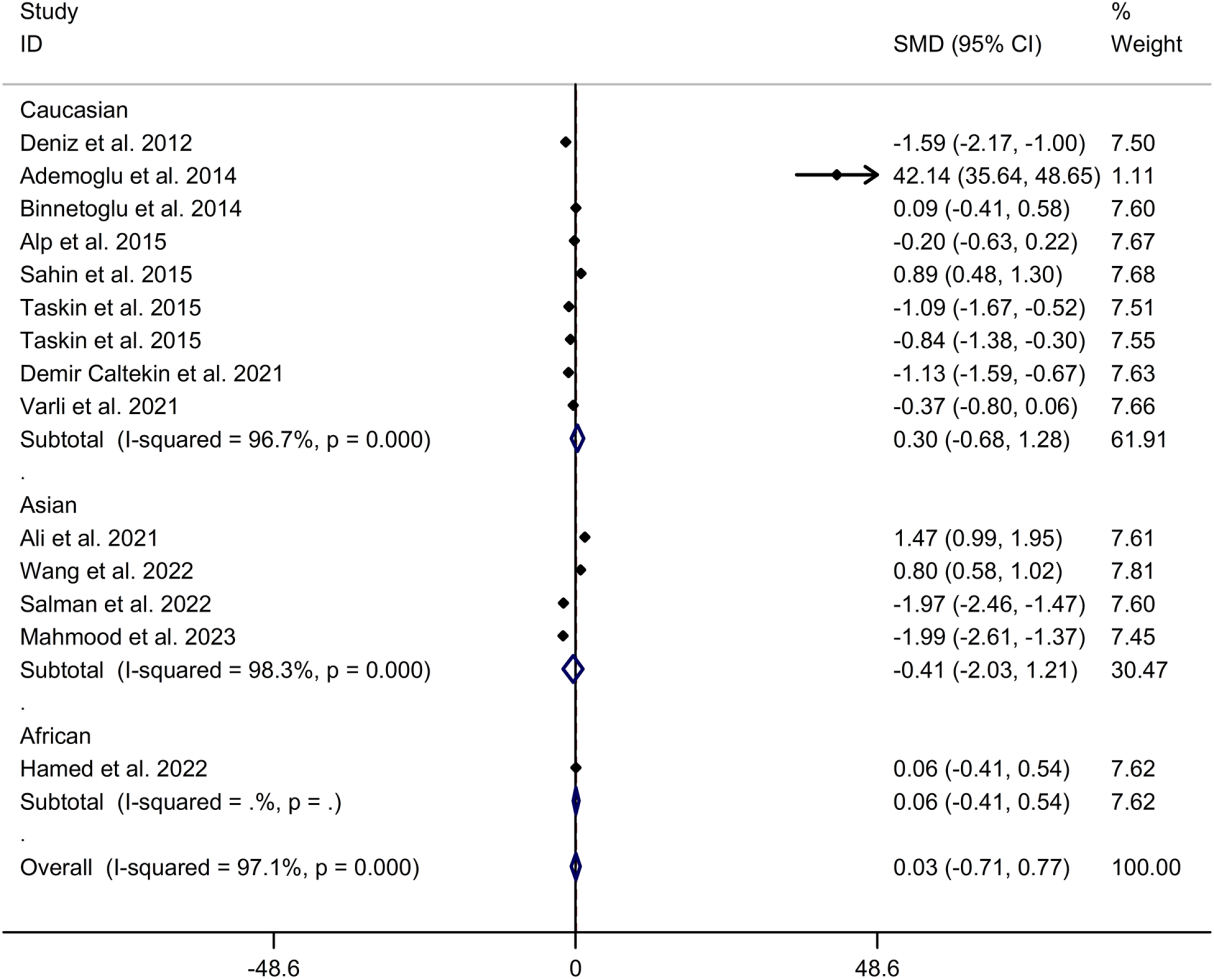
Subgroup analysis for comparison in blood nesfatin-1 level between patients with PCOS and controls with different ethnicities. CI, confidence interval; PCOS, polycystic ovary syndrome; SMD, standard mean difference.

**Figure 4:**
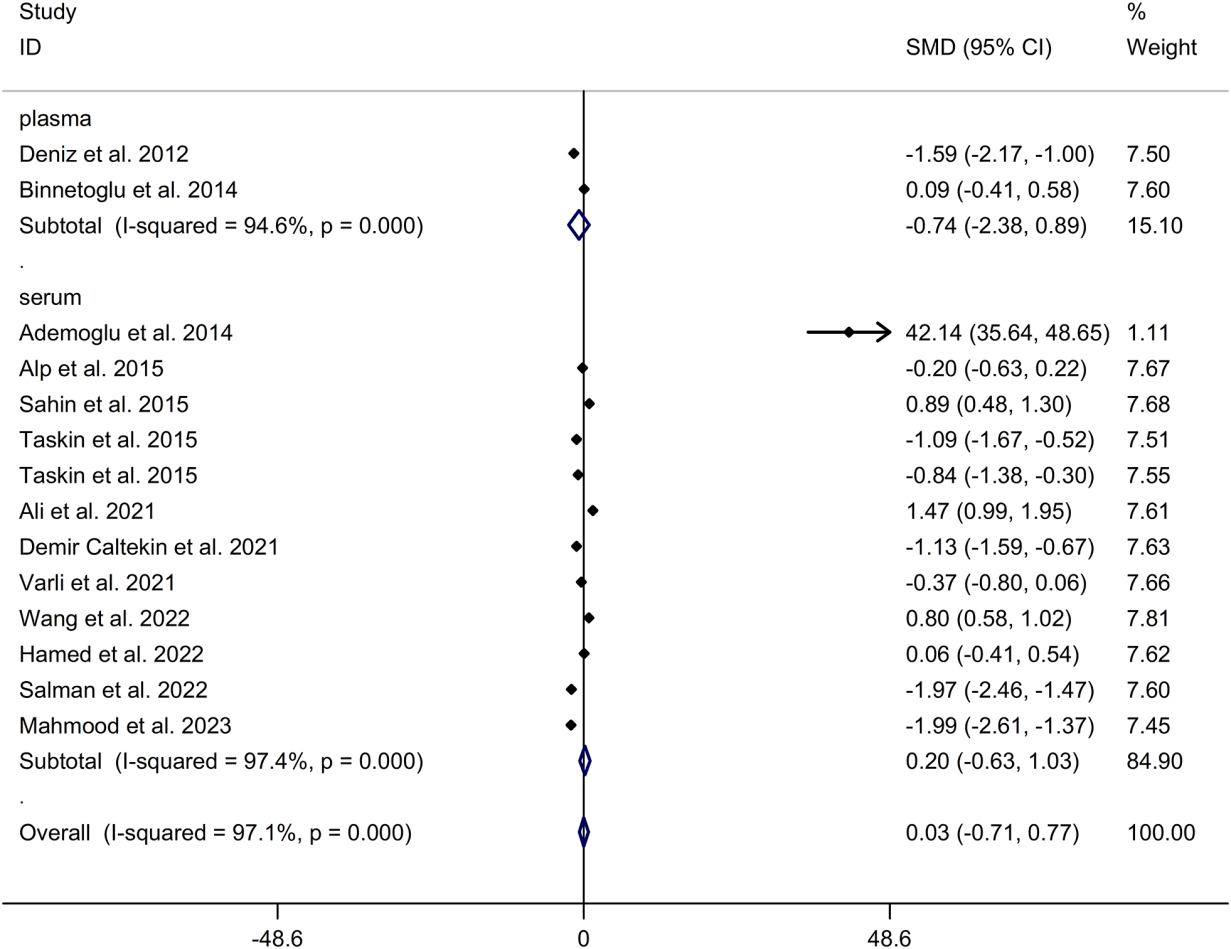
Subgroup analysis for comparison in nesfatin-1 level detected by different sample types between patients with PCOS and controls. CI, confidence interval; PCOS, polycystic ovary syndrome; SMD, standard mean difference.

**Figure 5:**
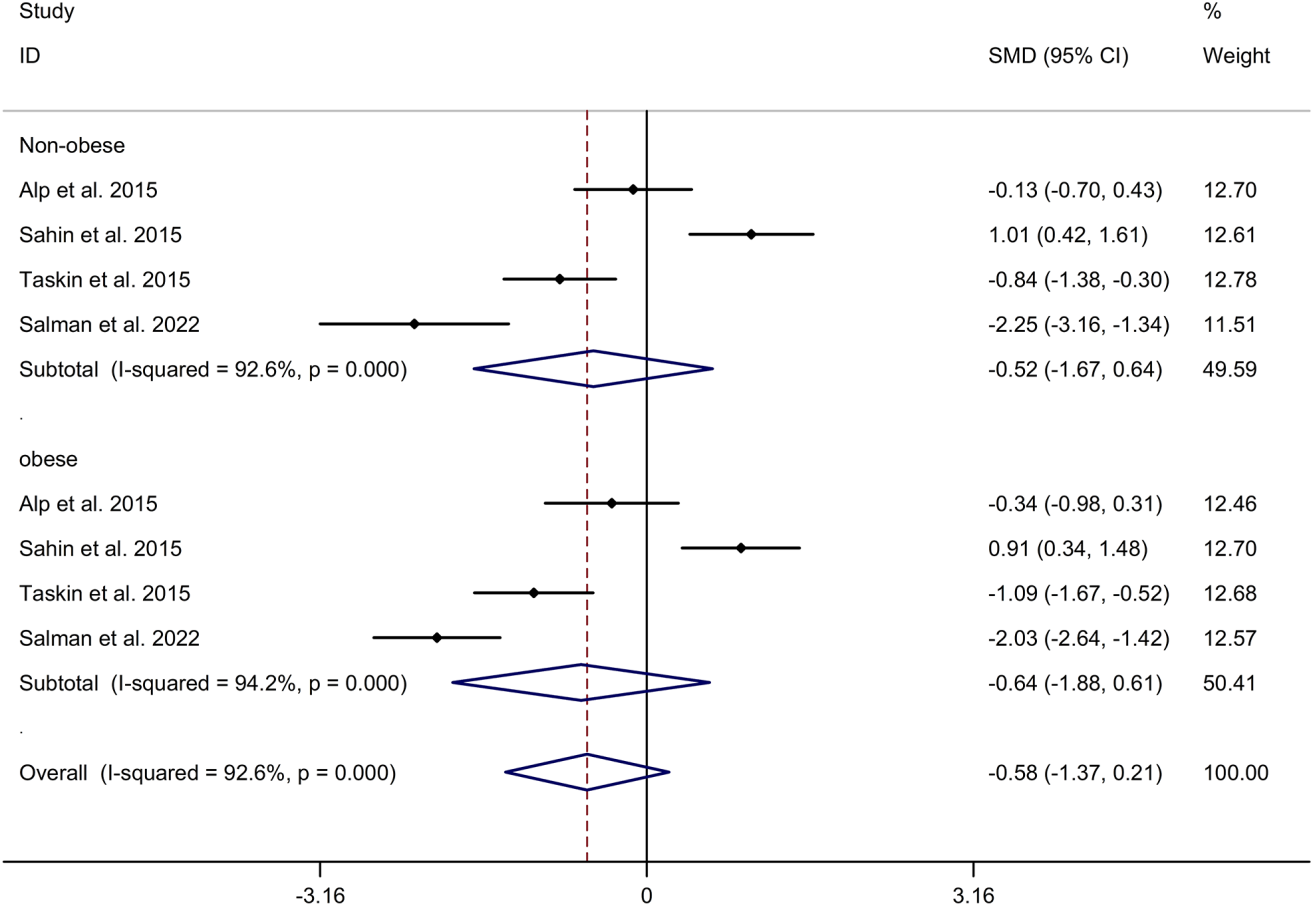
Subgroup analysis depending on presence and absence of obesity for comparison in nesfatin-1 level between patients with PCOS and controls. CI, confidence interval; PCOS, polycystic ovary syndrome; SMD, standard mean difference.

**Figure 6:**
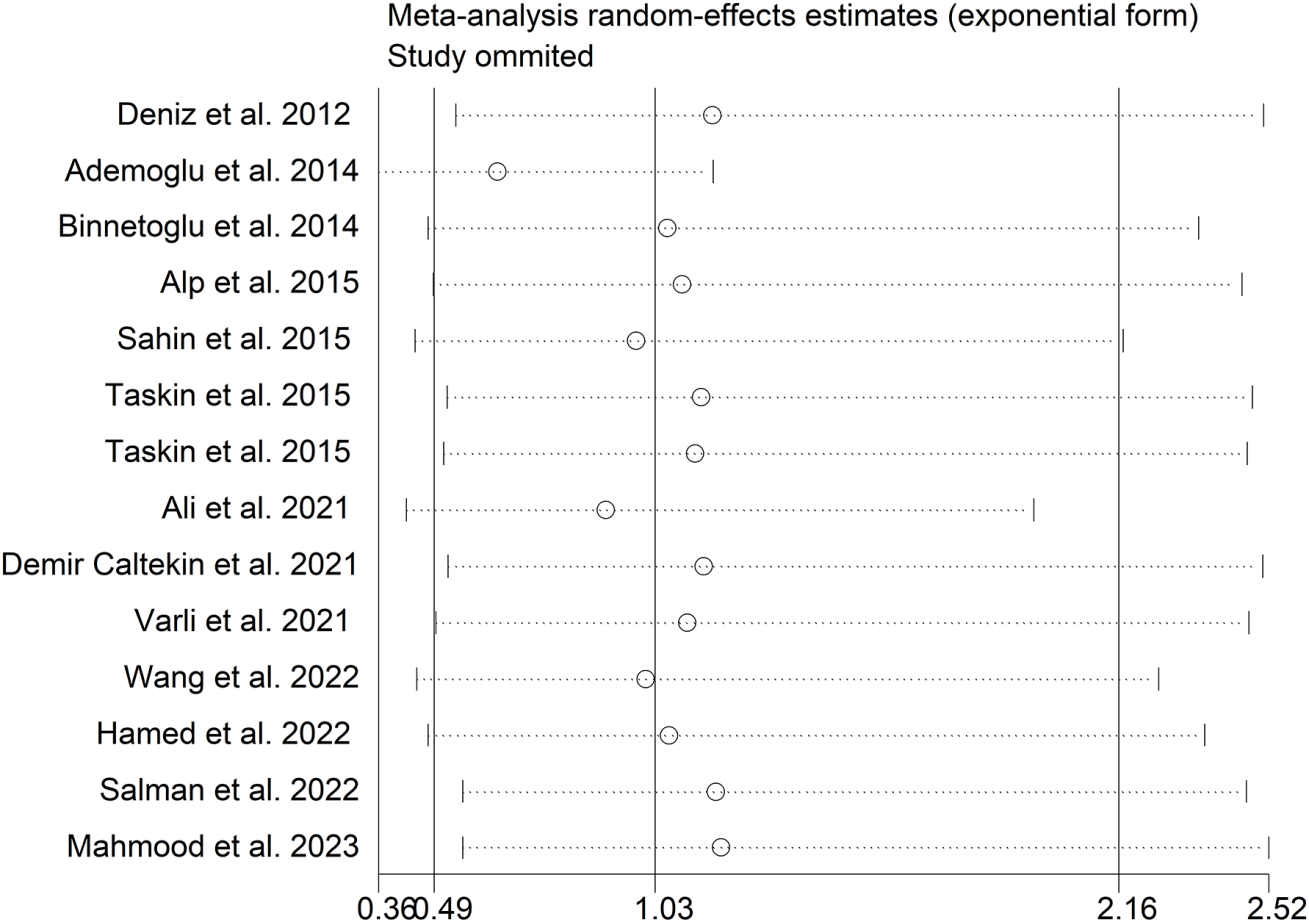
Sensitivity analysis for comparison in blood nesfatin-1 level between patients with PCOS and controls. PCOS, polycystic ovary syndrome.

**Figure 7:**
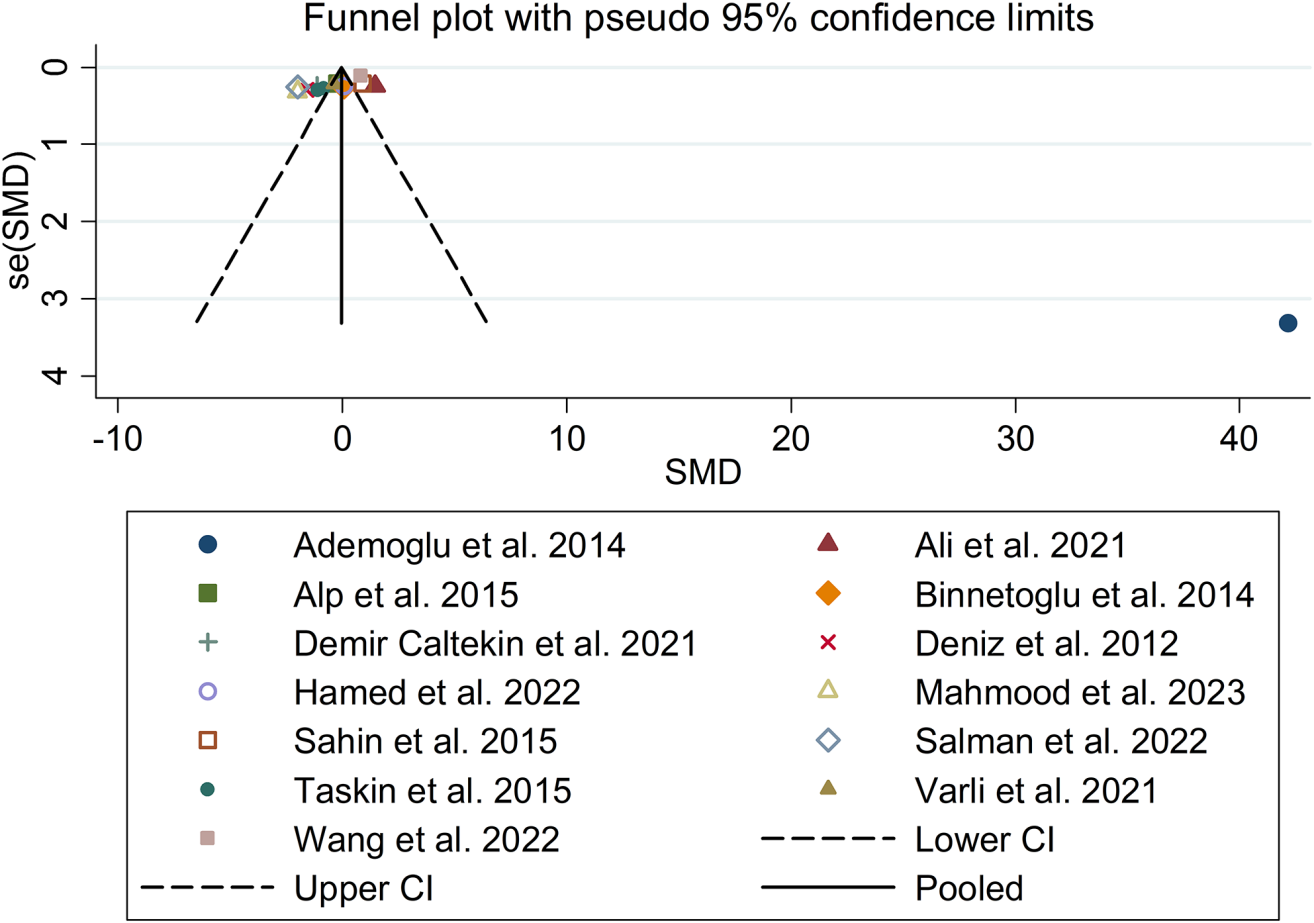
Funnel plot for comparison in blood nesfatin-1 level between patients with PCOS and controls. PCOS, polycystic ovary syndrome.

The present meta-analysis showed no significant difference in blood nesfatin-1 level between patients with PCOS and controls with a random effects model (SMD = 0.03; 95%CI: -0.71, 0.77; I^2^ = 97.1%, *p* value for Q test < 0.001; **Figure 2**). Meta-regression analysis showed that publication year and age were not responsible for heterogeneity across studies (publication year: *p* value = 0.369; age: *p* value = 0.632). Subgroup analysis for different ethnicities reported no significant difference in blood nesfatin-1 level between patients with PCOS and controls in both Caucasian and Asian populations (Caucasian: SMD = 0.30; 95%CI: -0.68, 1.28; Asian: SMD = -0.41; 95%CI: -2.03, 1.21; **Figure 3**). Subgroup analysis for different sample types reported no significant difference in serum nesfatin-1 level between patients with PCOS and controls (SMD = 0.20; 95%CI: -0.63, 1.03; **Figure 4**). Subgroup studies depending on presence and absence of obesity reported no significant difference in blood nesfatin-1 level between PCOS and controls in both obese and non-obese populations (**Figure 5**). Sensitivity analysis reported no changes in the direction of effect when any one study was excluded (**Figure 6**). Begg’s test and funnel plots showed no significant risk of publication bias (Begg’s test: *p* = 0.125; **Figure 7**).

## 4 Discussion

Our literature search yielded a comprehensive selection of 13 studies involving a substantial cohort of approximately 1300 participants, which enabled us to obtain more precise and potentially more accurate estimates of standardized mean differences (SMD) compared to the individual primary studies. Additionally, this extensive pool of data provided us with the opportunity to explore the potential factors contributing to any observed heterogeneity among these studies. Our literature search yielded a comprehensive selection of 13 studies involving a substantial cohort of approximately 1300 participants, which enabled us to obtain more precise and potentially more accurate estimates of SMD compared to the individual primary studies. Additionally, this extensive pool of data provided us with the opportunity to explore the potential factors contributing to any observed heterogeneity among these studies (SMD = 0.03; 95%CI: -0.71, 0.77). Furthermore, subgroup analysis revealed no differences in nesfatin-1 levels between Caucasian and Asian populations suffering from PCOS (Caucasian: SMD = 0.30; 95%CI: -0.68, 1.28; Asian: SMD = -0.41; 95%CI: -2.03, 1.21). PCOS cases exhibit a variable phenotypic spectrum, and previous studies have suggested that nesfatin-1 has effects on obesity (29, 30). Therefore, nesfatin-1 levels in PCOS may vary depending on the presence or absence of obesity. However, our present study reported no significant difference in blood nesfatin-1 levels between PCOS and controls in both obese and non-obese populations. Salman et al. (27) reported a sensitivity 93.5%, specificity of 79% and accuracy of 86.2% for serum nesfatin -1 level as predictor of PCOS using receiver operating characteristic (ROC) curve analysis. More studies were essential to explore the change of blood nesfatin-1 levels in PCOS.

Nesfatin-1, a peptide derived from the precursor nucleobindin2 (NUCB2), plays a significant role in regulating feeding behavior and energy metabolism (31). The etiology of PCOS involves multiple aspects, including ovarian and adrenal hyperandrogenism, neuro-endocrine and hypothalamic-pituitary dysfunction, disorders of peripheral insulin resistance, and overweight or obesity (32, 33). Many studies have suggested that nesfatin-1 has a direct influence on obesity, including food intake, glucose metabolism, weight loss, and cardiac functions (22, 34). A study by Algual et al. reported lower serum nesfatin-1 levels in individuals with metabolic syndrome compared to the control group (35). However, other studies have shown that serum nesfatin-1 concentrations were significantly lower in obese subjects compared to non-obese subjects (36, 37). These inconsistent results may contribute to the lack of significant difference in blood nesfatin-1 levels between PCOS subjects and controls. It has been observed to have an anorexic effect by reducing meal frequency and increasing the time interval between meals (19). In a study involving PCOS model rats, it was found that nesfatin-1 serum levels decreased significantly compared to the normal control group (38). These results were consistent with the analysis of ovarian nesfatin-1 mRNA and protein levels using RT-PCR and western blot techniques (38). Additionally, the study revealed a positive correlation between nesfatin-1 and follicle-stimulating hormone (FSH), estradiol (E_2_), and progesterone (P) (38). This suggests that the decrease in nesfatin-1 levels in PCOS may disrupt follicular cell development through the inhibition of FSH in folliculogenesis (38). For PCOS patients, elevated serum nesfatin-1 concentrations were directly associated with serum levels of prolactin (26). This may be attributed to the co-localization of nesfatin-1 and prolactin-releasing peptide producing neurons in adrenal medullary A1 and A2 catecholamine cell groups, as well as the co-expression of nesfatin-1 and prolactin-releasing peptide (39, 40). However, it is worth noting that some studies have reported the opposite findings. A previous study demonstrated that intravenous injection of nesfatin-1 significantly decreased blood sugar in hyperglycemic db/db mice, indicating that nesfatin-1 has hypoglycemic effects by accelerating insulin secretion through increased calcium ion influx via L-type channels in mouse pancreas islet beta-cells (41, 42). Caltekin et al. also revealed lower nesfatin-1 levels in PCOS patients compared to healthy individuals, suggesting that PCOS shares similarities with diabetes and gestational diabetes mellitus (GDM) due to weight and insulin resistance (23). A separate Japanese study provided evidence supporting a relationship between nesfatin-1 and the insulin receptor (43). However, further research is necessary to elucidate the precise mechanisms underlying the association between nesfatin-1 and PCOS.

In the current meta-analysis, several limitations should be acknowledged. Firstly, the number of included studies was limited, comprising only 13 studies, and most of these studies had small sample sizes. Secondly, the study solely focused on articles published in the English language, potentially introducing bias. This exclusion of non-English literature may have restricted the generalizability of the findings. Thirdly, complete access to detailed data sets, including potential confounders such as BMI, fasting blood glucose, insulin levels, homeostasis model assessment-insulin resistance (HOMA-IR) index, luteinizing hormone (LH), follicle stimulating hormone (FSH), smoking, and physical activity, was not available. These confounders may have influenced the results.

From this meta-analysis, it is concluded that there is no significant relationship between blood nesfatin-1 levels and PCOS. However, the precise role of nesfatin-1 in the pathogenesis of PCOS remains poorly understood. Consequently, further examination of our findings necessitates additional prospective evidence-like clinical studies.

## Data availability statement

The original contributions presented in the study are included in the article/**Supplementary Material**. Further inquiries can be directed to the corresponding authors.

## Author contributions

MW: Data curation, Formal Analysis, Investigation, Writing – original draft. JT: Formal Analysis, Investigation, Methodology, Project administration, Writing – original draft. QZ: Investigation, Methodology, Writing – review & editing. HT: Formal Analysis, Investigation, Methodology, Writing – review & editing. LT: Investigation, Writing – original draft, Writing – review & editing.
