# Supplementary material for: Blood nesfatin-1 levels in patients with polycystic ovary syndrome: a systematic review and meta-analysis
Source: Front Endocrinol (Lausanne). 2024 Jan 24;14:1275753. doi: 10.3389/fendo.2023.1275753 (PMC10847586; doi:10.3389/fendo.2023.1275753)
Supplement: Supplementary file 1 [file DataSheet_1.pdf]

MOOSE (Meta-analyses of Observational Studies in Epidemiology) Checklist

Item No	Recommendation	Reported on Page Number/Line Number	Reported on Section/Paragraph
**Reporting of Background**			
1	Problem definition	2-3/41-61	Introduction/1-2
2	Hypothesis statement	3/62-67	Introduction/3
3	Description of Study Outcome(s)	3/65-67	Introduction/3
4	Type of exposure or intervention used	3/65-67	Introduction/3
5	Type of study design used	3/65-67	Introduction/3
6	Study population	3/65-67	Introduction/3
**Reporting of Search Strategy**			
7	Qualifications of searchers (eg, librarians and investigators)	3/76	Methods/1
8	Search strategy, including time period included in the synthesis and keywords	3/76-81	Methods/1
9	Effort to include all available studies, including contact with authors	3/76-81	Methods/1
10	Databases and registries searched	3/76-81	Methods/1
11	Search software used, name and version, including special features used (eg, explosion)	3/76-81	Methods/4
12	Use of hand searching (eg, reference lists of obtained articles)	N/A we did not use hand [illegible]	N/A we did not use hand [illegible]
13	List of citations located and those excluded, including justification	N/A the study did not [illegible]	N/A the study did not [illegible]
14	Method for addressing articles published in languages other than English	N/A All articles were [illegible]	N/A All articles were [illegible]
15	Method of handling abstracts and unpublished studies	figure 1	figure 1
16	Description of any contact with authors	N/A the study showed no [illegible]	N/A the study showed no [illegible]

**Reporting of Methods**			
17	Description of relevance or appropriateness of studies assembled for assessing the hypothesis to be tested	3/84-86	Methods/2
18	Rationale for the selection and coding of data (eg, sound clinical principles or convenience)	3/90-93	Methods/3
19	Documentation of how data were classified and coded (eg, multiple raters, blinding, and interrater reliability)	3/90-93	Methods/3
20	Assessment of confounding (eg, comparability of cases and controls in studies where appropriate)	N/A we did not include [illegible]	N/A we did not include [illegible]
21	Assessment of study quality, including blinding of quality assessors; stratification or regression on possible predictors of study results	N/A we did not include [illegible]	N/A we did not include [illegible]
22	Assessment of heterogeneity	3-4/98-102	Methods/4
23	Description of statistical methods (eg, complete description of fixed or random effects models, justification of whether the chosen models account for predictors of study results, dose-response models, or cumulative meta-analysis) in sufficient detail to be replicated	3-4/95-106	Methods/4
24	Provision of appropriate tables and graphics	10-20	Figures and Table S1
**Reporting of Results**			
25	Graphic summarizing individual study estimates and overall estimate	4/118-121 11/Figure 2	Results/2 Figure 2
26	Table giving descriptive information for each study included	4/110-111 14-20/Table S1	Results/1 Table S1
27	Results of sensitivity testing (eg, subgroup analysis)	4/121-125 11-12/Figures [illegible]	Results/2 Figures 2-4
28	Indication of statistical uncertainty of findings	N/A the study did not	N/A the study did not
**Reporting of Discussion**			
29	Quantitative assessment of bias (eg, publication bias)	4/125-127 13/Figure 5	Results/2 Figure 5
30	Justification for exclusion (eg, exclusion of non–English-language citations)	N/A the study did not [illegible]	N/A the study did not [illegible]
31	Assessment of quality of included studies	N/A the study did not [illegible]	N/A the study did not [illegible]
**Reporting of Conclusions**			
32	Consideration of alternative explanations for observed results	5/165-166	Conclusions/1
33	Generalization of the conclusions (ie, appropriate for the data presented and within the domain of the literature review)	5/165-166	Conclusions/1
34	Guidelines for future research	5/166-168	Conclusions/1
35	Disclosure of funding source	6/172	Acknowledgments/1

From: Stroup DF, Berlin JA, Morton SC, *et al.*, for the Meta-analysis Of Observational Studies in Epidemiology (MOOSE) Group. Meta-analysis of Observational Studies in Epidemiology. A Proposal for Reporting. JAMA. 2000;283(15):2008-2012. doi: 10.1001/jama.283.15.2008.

Article information: https://dx.doi.org/10.21037/tcr-22-690
*As the checklist was provided upon initial submission, the page number/line number reported may be changed due to copy editing and may not be referable in the published version. In this case, the section/paragraph may be used as an alternative reference.
